# Early social gaze fixation and later inattention and hyperactivity–impulsivity symptoms in children: A longitudinal population‐based cohort study

**DOI:** 10.1002/pcn5.70380

**Published:** 2026-07-23

**Authors:** Masatsugu Orui, Mami Ishikuro, Taku Obara, Keiko Murakami, Aoi Noda, Genki Shinoda, Hirohito Metoki, Masahiro Kikuya, Naoki Nakaya, Tomoko Nishimura, Keiko Tanaka, Yoshihiro Miyake, Atsushi Hozawa, Kenji J. Tsuchiya, Shinichi Kuriyama

**Affiliations:** ^1^ International Research, Institute of Disaster Science Tohoku University Sendai Japan; ^2^ Tohoku Medical Megabank Organization Tohoku University Sendai Japan; ^3^ Graduate School of Medicine Tohoku University Sendai Japan; ^4^ Tohoku University Hospital Tohoku University Sendai Japan; ^5^ Graduate School of Medicine The University of Tokyo Tokyo Japan; ^6^ Division of Public Health, Hygiene and Epidemiology Tohoku Medical and Pharmaceutical University Sendai Japan; ^7^ Department of Hygiene and Public Health Teikyo University School of Medicine Tokyo Japan; ^8^ United Graduate School of Child Development The University of Osaka, Kanazawa University, Hamamatsu University School of Medicine, Chiba University, and University of Fukui Suita Japan; ^9^ Department of Epidemiology and Public Health Ehime University Graduate School of Medicine Toon Japan; ^10^ Research Center for Child Mental Development Hamamatsu University School of Medicine Hamamatsu Japan

**Keywords:** ADHD, ASD, eye‐tracking, Gazefinder®, inattention, longitudinal

## Abstract

**Aim:**

To examine whether gaze patterns related to ASD are also associated with later inattention and hyperactivity–impulsivity symptoms and whether such gaze patterns may reflect broader neurodevelopmental vulnerability.

**Methods:**

Using data from the Tohoku Medical Megabank Project Birth and Three‐Generation Cohort Study, we examined whether gaze fixation on social information at age 4 was longitudinally associated with inattention and hyperactivity–impulsivity symptoms at age 8. Of 6571 children with gaze data, 3043 whose caregivers completed the ADHD Rating Scale (ADHD‐RS) were included. Social gaze—defined as fixation on the eyes in human‐face stimuli and on people in people–geometry stimuli—was measured using Gazefinder®. Percentile scores for inattention and hyperactivity–impulsivity were analyzed separately for boys and girls using generalized linear models.

**Results:**

In boys, shorter gaze fixation on social information at age 4 was significantly associated with higher inattention and hyperactivity–impulsivity percentiles at age 8. In girls, no significant associations were observed in the primary complete‐case analyses after full adjustment; however, significant associations for the small window stimulus emerged after inverse‐probability weighting.

**Conclusion:**

Reduced social gaze at age 4, measured using an eye‐tracking system originally developed for ASD, was associated with later inattention and hyperactivity–impulsivity symptoms. These findings suggest that early alterations in social gaze may represent a developmental characteristic associated with broader neurodevelopmental vulnerability and later behavioral symptoms, including inattention and hyperactivity–impulsivity.

## Graphical abstract

Reduced social gaze fixation at age 4 was associated with increased inattention and hyperactivity–impulsivity symptoms at age 8 among 3043 children. Early ASD‐oriented eye‐tracking measures may help identify children at risk for later inattention and hyperactivity–impulsivity symptoms.

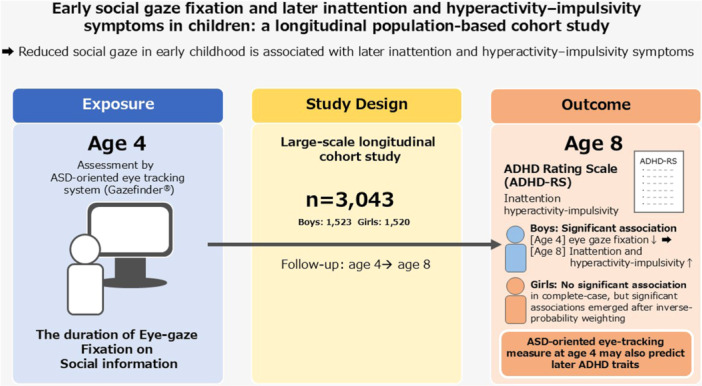

## BACKGROUND

Autism spectrum disorder (ASD) is a neurodevelopmental disorder characterized by difficulties with social interaction and communication as well as restricted interests and repetitive behavior, and those with the disorder exhibit biases in social attention from an early developmental stage.^1^ ASD often coexists with attention‐deficit/hyperactivity disorder (ADHD), which has inattention and hyperactivity‐impulsivity as main features.^2^, ^3^ An increasing number of studies seek to understand ASD and ADHD as a continuum and from a dimensional framework rather than as separate entities based on changes in Diagnostic and Statistical Manual of Mental Disorders, Fifth Edition (DSM‐5) that now allow for a combined diagnosis of the disorders.^4^, ^5^, ^6^ In addition to these disorders partially overlapping in neurodevelopmental basis (e.g., attentional control, social cognition, and executive function), they may also share behavioral characteristics such as bias in social attention and difficulties with maintaining interest in others.^7^

Studies have consistently reported that the duration of eye‐gaze fixation on social information such as the face and eyes of others is short in ASD patients.^8^, ^9^, ^10^, ^11^, ^12^, ^13^, ^14^, ^15^, ^16^, ^17^ Research using eye tracking has advanced to capture this characteristic, with utility demonstrated for risk assessment and screening for ASD in early childhood^8^, ^9^, ^10^, ^11^, ^12^, ^13^, ^14^, ^15^, ^16^, ^17^ and reports that eye tracking systems such as Gazefinder® can be applied to clinical settings.^18^, ^19^, ^20^, ^21^, ^22^, ^23^, ^24^, ^25^, ^26^, ^27^ Studies examining the possibility of using eye tracking systems for children diagnosed with ADHD have also been published,^28^, ^29^, ^30^, ^31^ as well as studies reporting that these children experience difficulties with impulse control, working memory, and emotional and social information processing, and attention deficits during task performance.^32^ However, gaze analysis in conventional ADHD research has mainly focused on task‐related attentional control—the distribution of attention and wavering of gaze when carrying out tasks^28^, ^29^, ^30^, ^31^, ^32^, ^33^—with only a few studies directly assessing the duration of eye gaze fixation on social information such as human facial expressions among ADHD‐diagnosed children.^34^, ^35^ Moreover, to our knowledge, only one study used an eye‐tracking system to assess relationships between the duration of eye gaze fixation on social information and ADHD characteristics in individuals diagnosed with ASD,^35^ with no such studies targeting children.

We previously used Gazefinder®, an eye‐tracking system developed as an ASD screening tool, to demonstrate a potential relationship between the duration of eye gaze fixation on social information at age 4 and both peer problems (one of the core features of ASD) and hyperactivity‐inattention (one of the core features of ADHD).^36^ These findings suggest that alterations in early social gaze may be associated with multiple domains of later behavioral difficulties rather than being specific to a single disorder. However, that study assessed ADHD characteristics using a general behavioral rating scale (Strengths and Difficulties Questionnaire [SDQ]) rather than an ADHD‐focused symptom scale. Therefore, in the present study, we examined whether reduced early social gaze is longitudinally associated with later inattention and hyperactivity–impulsivity symptoms at age 8, as assessed using the ADHD Rating Scale (ADHD‐RS). By focusing on inattention and hyperactivity–impulsivity symptoms assessed using the ADHD‐RS, this analysis aimed to clarify whether gaze patterns related to ASD are also associated with ADHD‐focused symptoms and to further explore whether such gaze patterns may reflect broader neurodevelopmental vulnerability. We hypothesized that reduced gaze fixation on social information in early childhood (age 4) would be longitudinally associated with later inattention and hyperactivity–impulsivity symptoms at age 8.

## METHODS

### TMM BirThree Cohort Study and participants

The Tohoku Medical Megabank Project (TMM) aims to provide medical care to address the damage caused by the Great East Japan Earthquake in 2011 and to support health services through personalized medical care and assistance for affected residents by disaster. The Birth and Three‐Generation (BirThree) Cohort Study, a part of the TMM, recruited pregnant women, their children, partners, and parents from specific regions of Miyagi and Iwate Prefectures, Japan, between July 2013 and March 2017. Details regarding the recruitment process have been described previously^37^, ^38^

**Figure 1:**
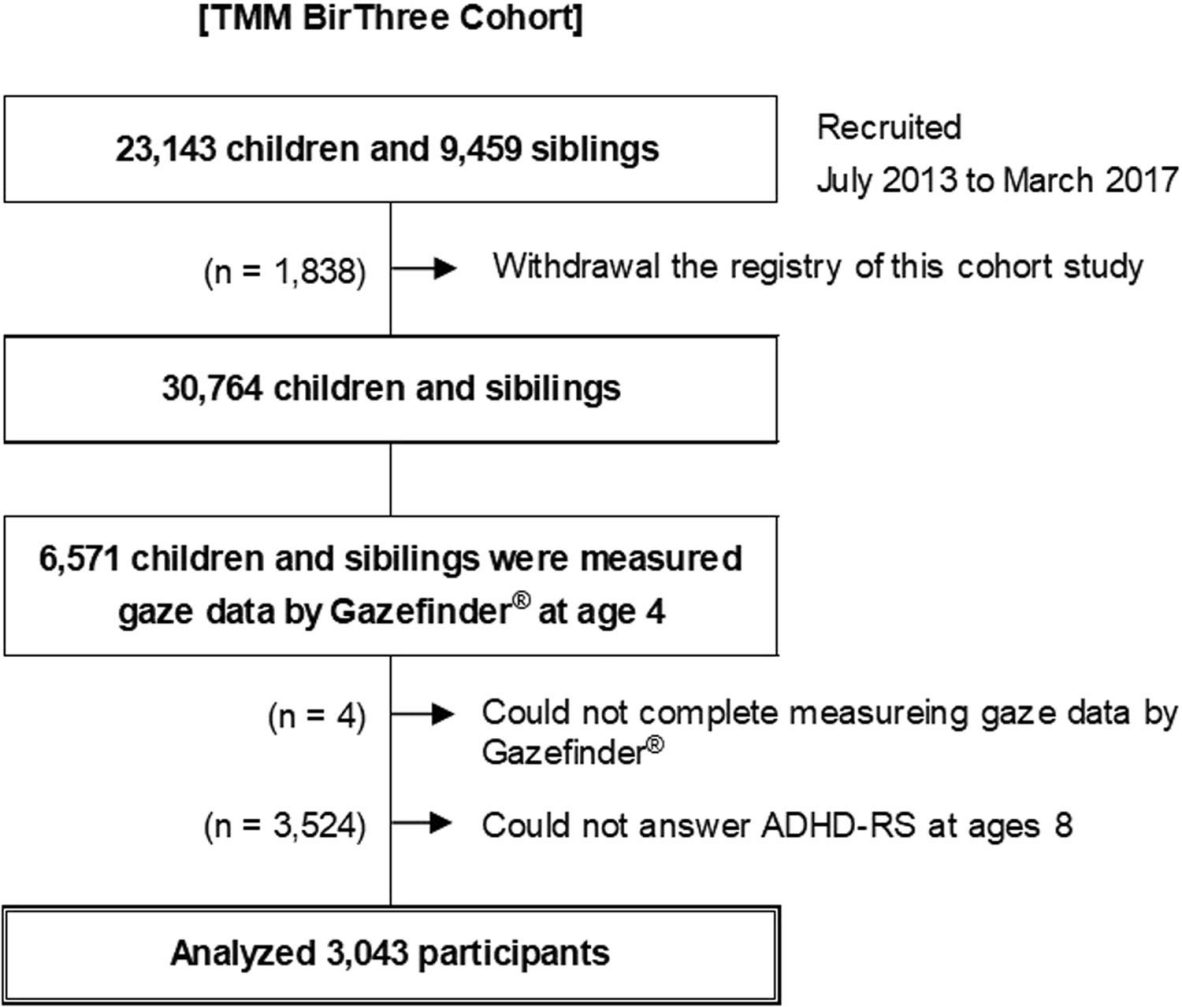
Participants of the TMM BirThree cohort and analyzed subjects. ADHD‐RS, attention‐deficit/hyperactivity disorder Rating Scale; TMM, Tohoku Medical Megabank Project.

A total of 23,143 children and 9459 siblings are registered in the BirThree Cohort Study, among which 6571 underwent gaze data measurements using Gazefinder® at age 4. Four children did not complete gaze measurements for all stimuli using Gazefinder®. We included in the analysis children who both answered the ADHD‐Rating Scale (ADHD‐RS) at age 8 in a follow‐up study^38^ and for whom gaze data at age 4 were available. The final study population consisted of 3043 children (Figure 1). Differences between participants who were followed up and those lost to follow‐up were examined using baseline characteristics and gaze indices.

The study focused on children at age 4 because all children in Japan undergo a health checkup at 3 years and 6 months of age. This timeline aligns with the possibility of adopting gaze measurement as part of health checkups in the future.

### Gazefinder® measurements

Participants sat in front of a monitor at a distance of 60 cm under the direction of the study coordinator, and gaze measurements were taken using Gazefinder® for a period of about 2 min. The position of the eye was measured using a camera with an infrared light source located at the bottom of a 19‐inch monitor (1280 × 1024 pixels). The position of the eye was recorded based on the corneal reflex, and *x* and *y* coordinates were obtained at a 50 Hz frequency. Before gaze measurements, eye position calibrations were performed. A feature of this eye‐tracking system is that the assessment ends after simply watching a stimulus image displayed on the monitor for about 2 min.

**Figure 2:**
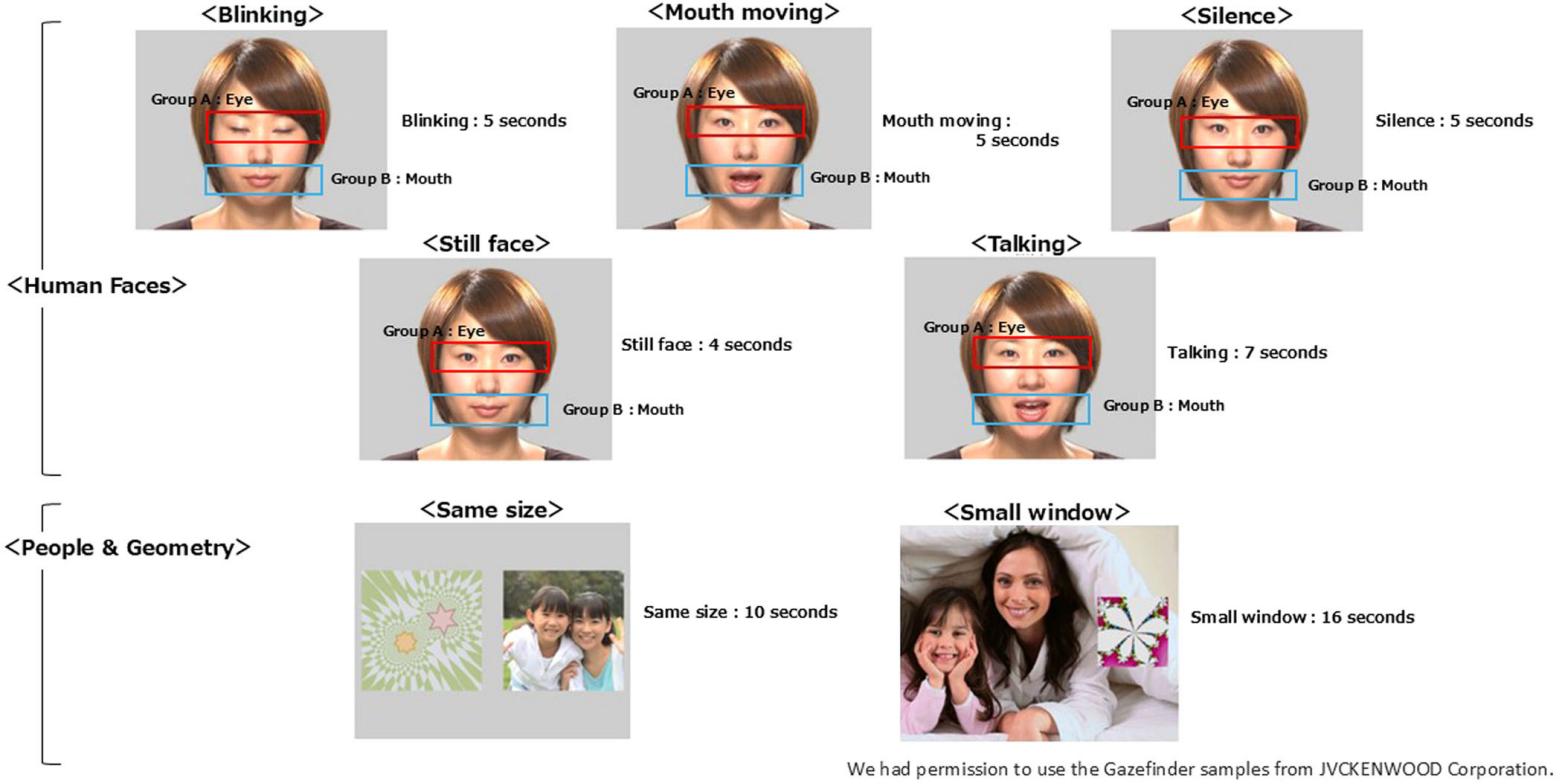
Gazefinder's five stimuli of human faces and two stimuli of people & geometry.

Following calibration, eight short videos were presented. These included five videos which displayed specific human facial expressions or movements: blinking (5 s), mouth moving (5 s), silence (5 s), still face (4 s), and talking (7 s). In addition, two stimulus videos that displayed people and geometry were presented: a 10‐s video of people and geometry displayed at the same size (“same size”), and a 16‐s video of a small window displaying geometry on top of a human image (“small window”) (Figure 2).

We defined the proportion of time the gaze was fixed on a person's eye in response to the five stimuli involving human faces, as well as the time the gaze was fixed on people in the people and geometry stimuli as “eye gaze fixation on social information”.^13^ We measured and analyzed the proportion of time the gaze was fixed on these targets. The research framework, including the use of Gazefinder®, of the TMM BirThree Cohort Study has been reported previously.^39^

### Measurement of Inattention and Hyperactivity–impulsivity using the ADHD‐RS

ADHD‐RS, developed by DuPaul et al. and consisting of 18 questions relating to a child's behavior in the past 6 months, is widely used to support a diagnosis of ADHD in children aged 5–17 years.^40^ It is a self‐reported assessment answered by parents or teachers that measures the frequency of behaviors, with the options of whether the behavior occurs “always or often,” “often,” “somewhat,” and “rarely or never.” Contents of the survey include items relating to inattention and hyperactivity‐impulsivity traits.

The ADHD‐RS has been translated into Japanese and validated.^41^ In the present study, we used the parent version of the survey. Moreover, rather than raw scores, we conducted analyses using percentile values based on the general Japanese population^42^ by referring to prior research.^43^

At age 8, caregivers completed the ADHD‐RS questionnaire at the cohort study center as part of the follow‐up assessment.

### Information on doctor‐diagnosed neurodevelopmental disorders

Information on doctor‐diagnosed ASD and ADHD by age 7 was collected through follow‐up survey questionnaires from caregiver.

### Covariates

Covariates in the analysis included the birth weight, mother's age at birth, and mother's educational attainment (up to high school or more).

### Statistical analysis

#### Categorized gaze data at age 4

Referencing the previous study,^15^ we analyzed the duration of eye gaze fixation on the monitor in response to two different types of stimuli—human facial stimuli (blinking, mouth moving, silence, still face, and talking) and people and geometry stimuli (same size and small window). First, we analyzed the distribution of the duration of eye gaze fixation in response to each stimulus. Given concerns regarding the reliability of gaze data for children who spent most of the time focused outside the monitor, we excluded from the analysis 178 children with a duration of gaze fixation that was less than the 5th percentile of the total cohort.

#### Association between gaze data at age 4 and ADHD‐RS score at age 8

First, we obtained general characteristics including the child's sex, birth weight, mother's age at birth, and mother's educational attainment, followed by the distribution of scores for each item relating to inattention and hyperactivity‐impulsivity traits in the ADHD‐RS. Finally, associations between gaze fixation on social information measured using Gazefinder® at age 4 and ADHD‐RS inattention and hyperactivity–impulsivity scores at age 8 were examined using generalized linear models (GLMs) with a gamma distribution. Model 1 adjusted for child's birth weight, and Model 2 adjusted for child's birth weight, mother's age at birth, and mother's educational attainment. Analyses were conducted separately for boys and girls.

To assess the potential impact of attrition bias, we conducted a sensitivity analysis using inverse‐probability weighting (IPW), including both participants who completed and those who did not complete the ADHD‐RS. The weighting model included child sex, birth weight, maternal age at delivery, maternal educational attainment, and all seven gaze indices measured by Gazefinder® at age 4.

### Overall statistical analysis

All statistical analyses were performed using Stata version 19 (StataCorp LLC, College Station, TX, USA).

### Ethical considerations

This study was approved by the ethics committee of the Tohoku University School of Medicine (approval: 2013‐4‐103; date: May 10, 2013). All participants in the TMM BirThree Cohort Study provided informed consent. As all participants in this study were minors, their guardians provided proxy consent. Finally, this study was conducted in compliance with the Declaration of Helsinki, as it involved human participants.

## RESULTS

### Basic characteristics and doctor‐diagnosed neurodevelopmental disorders

There was a total of 3043 participants, with about an equal number of boys and girls (1523 boys and 1520 girls). Mean birth weight (± standard deviation (SD)) was 3046 g (±442.8) for boys and 2964 g (±433.1) for girls. There were no major differences in mother's age at birth for boys and girls, with mothers giving birth in the 30 s accounting for about two‐thirds of participants. Similarly, for mother's educational attainment, the proportion of mothers who went to vocational school, junior college, college, or graduate school was about 69% for both boys and girls (Table 1).

**Table 1:** Basic characteristics, ASHD‐RS score, doctor‐diagnosed neurodevelopmental disorders and Gazefinder® indices.

	Boys (*n* = 1523)		Girls (*n* = 1520)		Total (*n* = 3043)	
Birth weight						
Mean (g)	3046	442.8	2964	433.1	3003	444.9
Mother's age						
10 ages	4	0.3%	3	0.2%	7	0.2%
20 ages	400	26.3%	386	25.4%	786	25.8%
30 ages	1024	67.2%	997	65.6%	2,021	66.4%
40 age or more	95	6.2%	134	8.8%	229	7.5%
Mother's educational attainment						
Up to high school	413	31.0%	390	30.2%	803	30.6%
More than high school	919	69.0%	900	69.8%	1819	69.4%
ADHD‐RS score (mean, SD)						
Inattention	6.34	5.39	4.49	4.47	5.44	5.04
Hyperactivity‐impulsivity	3.75	4.12	2.19	2.88	2.97	3.64
ADHD‐RS percentile (93%tile or higher)						
Inattention (*n*, %)	102	6.7%	73	4.8%	175	5.8%
Hyperactivity‐impulsivity (*n*, %)	32	2.1%	66	4.3%	98	3.2%
Doctor‐diagnosed neurodevelopmental disorder						
ASD and ADHD (*n*, %)	4	0.3%	0	0.0%	4	0.1%
Gazefinder® indices (mean ± SD)						
Blinking	0.683	0.208	0.697	0.194	0.690	0.201
Mouth moving	0.223	0.147	0.232	0.148	0.227	0.148
Silence	0.360	0.220	0.379	0.215	0.369	0.218
Still face	0.509	0.209	0.519	0.202	0.514	0.206
Talking	0.260	0.167	0.264	0.156	0.262	0.162
Same size	0.441	0.441	0.504	0.148	0.472	0.152
Small window	0.415	0.415	0.487	0.163	0.450	0.165

Abbreviation: SD, standard deviation.

For the ADHD‐RS, boys had higher mean (± SD) scores for inattention and hyperactivity‐impulsivity traits (inattention: 6.34 (± 5.39) for boys and 4.49 (± 4.47) for girls; hyperactivity‐impulsivity: 3.75 (± 4.12) for boys and 2.19 (± 2.88) for girls). The number of boys in the 93rd percentile or higher (considered optimal for ADHD screening) for inattention and hyperactivity‐impulsivity traits were 102 (6.7%) and 32 (2.1%), respectively; corresponding numbers for girls were 73 (4.8%) and 66 (4.3%), respectively. Among the participants, only four children (0.1%) were diagnosed by a doctor by age 8 (Table 1).

Baseline characteristics and gaze indices were generally comparable between participants included in the analysis and those excluded because of missing ADHD‐RS data, with the exception of the Gazefinder® blinking stimulus (Supporting Inforamtion S1: Table 1).

### Associations between gaze data at age 4 and ADHD‐RS score at age 8

In boys, the inattention score was significantly associated with still face, talking, same size, and small window stimuli in Models 1 and 2. In particular, the small window stimulus showed the strongest association in both Model 1 (*β* = −17.741; 95% confidence interval (CI): −28.067, −7.416; *p* < 0.001) and Model 2 (*β* = −21.017; 95% CI: −31.973, −10.060; *p* < 0.001). For girls, while same size and small window stimuli were significantly associated with the inattention score in Model 1 as with boys, significant associations were not observed in Model 2.

In boys, the hyperactivity‐impulsivity score was significantly associated with silence, still face, talking, same size, and small window stimuli in Model 2. In particular, associations with the small window stimulus were strong in both Model 1 (*β* = −17.077; 95% CI: −26.404, −7.750; *p* < 0.001) and Model 2 (*β* = −19.486; 95% CI: −29.224, −9.748; *p* < 0.001). For girls, while same size and small window stimuli were significantly associated with the hyperactivity‐impulsivity score in Model 1, significant associations were not observed in Model 2 (Tables 2 and 3).

**Table 2:** The duration of eye gaze fixation on social information at age 4 and ADHD‐RS percentile score at age 8 among boys (GLM).

Boys	ADHD‐RS Inattention at age 8						ADHD‐RS Hyperactivity‐impulsivity at age 8					
	Adjusted β*1	(95%CI)	*p* Value	Adjusted β*2	(95%CI)	*p* Value	Adjusted β*1	(95%CI)	*p*Value	Adjusted β*2	(95%CI)	*p* Value
[Human face]												
Blinking	−1.476	(−9.190, 6.238)	0.708	−0.559	(−8.897, 7.780)	0.896	−1.301	(−8.214, 5.611)	0.712	−1.585	(−9.068, 5.898)	0.678
Mouth moving	−2.311	(−13.150, 8.528)	0.676	−1.422	(−13.324, 10.479)	0.815	−3.751	(−13.022, 5.519)	0.428	−4.871	(−15.289, 5.547)	0.359
Silence	−3.810	(−11.499, 3.880)	0.332	−3.654	(−12.014, 4.705)	0.392	**−6.870**	**(−13.428, −0.311)**	**0.040**	**−7.994**	**(−15.039, −0.949)**	**0.026**
Still face	**−9.236**	**(−17.454, −1.017)**	**0.028**	**−10.849**	**(−19.696, −2.003)**	**0.016**	**−7.943**	**(−15.368, −0.519)**	**0.036**	**−8.874**	**(−16.800, −0.948)**	**0.028**
Talking	**−10.753**	**(−21.082, −0.424)**	**0.041**	**−12.804**	**(−23.895, −1.713)**	**0.024**	−7.979	(−17.028, 1.070)	0.084	**−10.245**	**(−19.804, −0.685)**	**0.036**
[People & Geometory]												
Same size	**−17.797**	**(−28.868, −6.726)**	**0.002**	**−19.912**	**(−31.656,−8.168)**	**0.001**	**−11.848**	**(−21.938, −1.759)**	**0.021**	**−15.444**	**(−26.082, −4.806)**	**0.004**
Small window	**−17.741**	**(−28.067, −7.416)**	**0.001**	**−21.017**	**(−31.973, −10.060)**	**<0.001**	**−17.077**	**(−26.404, −7.750)**	**<0.001**	**−19.486**	**(−29.224, −9.748)**	**<0.001**

*Note*: Bold: *p* < 0.05, Adjusted β1: Adjusted by birth weight, Adjusted β2: Adjusted by birth weight, mother's age, and mother's educational attainment.

**Table 3:** The duration of eye gaze fixation on social information at age 4 and ADHD‐RS percentile score at age 8 among girls (GLM).

	ADHD‐RS Inattention at age 8															ADHD‐RS Hyperactivity‐impulsivity at age 8													
Girls	Adjusted β*1	(95%CI)					*p* Value		Adjusted β*2	(95%CI)					*p* Value	Adjusted β*1	(95%CI)					*p* Value	Adjusted β*2	(95%CI)					*p*‐Value
[Human face]																													
Blinking	−5.180	(−12.760, 2.400)					0.180		0.196	(−8.134, 8.526)					0.963	−1.725	(−7.502, 4.052)					0.558	0.993	(−5.306, 7.292)					0.757
Mouth moving	−7.800	(−17.419, 1.820)					0.112		−6.938	(−17.384, 3.508)					0.193	4.653	(−3.281, 12.586)					0.250	4.087	(−4.464, 12.638)					0.349
Silence	−2.494	(−9.900, 4.913)					0.509		0.871	(−7.189, 8.931)					0.832	1.787	(−4.001, 7.576)					0.545	3.981	(−2.287, 10.249)					0.213
Still face	−1.494	(−8.991, 6.003)					0.696		2.012	(−6.141, 10.165)					0.629	−2.003	(−7.875, 3.870)					0.504	0.912	(−5.466, 7.290)					0.779
Talking	−3.121	(−12.227, 5.986)					0.502		−1.540	(−11.420, 8.340)					0.760	0.882	(−6.609, 8.373)					0.818	2.644	(−5.554, 10.843)					0.527
[People & Geometory]																													
Same size	**−17.461**	**(−27.973**, **−6.949)**					**0.001**		−9.167	(−20.725, 2.391)					0.120	**−8.224**	**(−16.321**, **−0.127)**					**0.047**	−3.022	(−11.825, 5.780)					0.501
Small window	**−14.228**	**(−23.448**, **−5.008)**					**0.002**		−8.833	(−18.864, 1.197)					0.084	**−11.672**	**(−19.196**, **−4.147)**					**0.002**	−7.699	(−15.786, 0.388)					0.062

*Note*: Bold: *p *< 0.05, Adjusted β1: Adjusted by birth weight, Adjusted β2: Adjusted by birth weight, mother's age, and mother's educational attainment

Furthermore, the overall direction of the associations remained consistent after the IPW. Although the primary complete‐case analyses did not show significant associations among girls after full adjustment, significant associations between the small window stimulus and both inattention and hyperactivity–impulsivity were observed in the IPW analyses (Supporting Information S2: Tables 2‐1 and 2‐2).

## DISCUSSION

The present large‐scale community‐based study revealed associations between shorter gaze fixation on social information at age 4 and inattention and hyperactivity–impulsivity traits at age 8 in boys, with a particularly strong association observed for the small window stimulus of the Gazefinder® in the primary complete‐case analyses. Although these associations were not significant among girls in the primary fully adjusted analyses, IPW analyses demonstrated significant associations between the small window stimulus and both inattention and hyperactivity–impulsivity symptoms among girls, while maintaining the overall direction of the observed associations.

We first consider on whether the findings of the present study can be generalized. The mean scores for inattention and hyperactivity‐impulsivity traits of the ADHD‐RS at age 8 for both boys and girls of the present study essentially matched the results of a large‐scale survey targeting community‐based elementary and junior high school students,^44^ suggesting that our participants are not a unique population.

Compared with our previous study (Ref. 38), the present study extends our previous findings in several important ways. Consistent with Ref. 38, the significant associations were observed for the small window social gaze stimulus, supporting the reproducibility of the association between reduced social gaze fixation and later emotional and behavioral symptoms. However, whereas Ref. 38 evaluated hyperactivity/inattention using the SDQ as a broad emotional and behavioral measure, the present study used the ADHD‐RS, which separately assesses inattention and hyperactivity/impulsivity symptom domains. Using this ADHD‐focused symptom scale, we demonstrated that reduced gaze fixation on the small window stimulus was associated with both inattention and hyperactivity/impulsivity symptoms. These findings suggest that the present study represents not merely a change in measurement instrument, but rather a replication and extension of our previous findings using a more symptom‐focused assessment tool.

Given that 3524 participants did not complete the ADHD‐RS assessment, there was concern regarding potential attrition bias. Therefore, we conducted a sensitivity analysis using IPW, including both participants who completed and those who did not complete the ADHD‐RS. As a result, the direction of the associations remained consistent with those observed in the primary complete‐case analyses. However, significant associations between the small window stimulus and both inattention and hyperactivity–impulsivity symptoms were observed among girls after IPW adjustment. Thus, the findings among girls should be interpreted in the context of the primary complete‐case analyses, in which the associations did not reach statistical significance (Supporting Information S2: Tables 2‐1 and 2‐2).

Then, we consider the mechanism underlying the particularly strong association with the small window stimulus. According to Musser et al., who assessed the influence of value and salience on attentional selection in elementary‐aged children (ages 6–12 years), those diagnosed with ADHD tended to be biased toward salience and high value stimuli rather than the face.^45^ In another study, children with a dual diagnosis of ASD and ADHD were reported to have a lower gaze fixation count to faces compared with typically developing children.^46^ These reports suggest that the ease of gaze fixation on conspicuous stimuli is a characteristic of ADHD which could explain our findings. On the other hand, it was unclear from previous studies whether gaze fixation on conspicuous stimuli is related to inattention or hyperactivity‐impulsivity traits, or both, in ADHD. However, our results suggest a stronger relationship with inattention traits. Further studies to elucidate the underlying mechanisms are warranted.

An important consideration is whether the gaze metric used in the present study reflects an ADHD‐specific marker or a broader indicator of neurodevelopmental vulnerability. In our previous study using the same cohort, shorter gaze fixation on social information at age 4 was associated not only with hyperactivity/inattention but also with emotional and peer problems assessed using the SDQ at ages 6–7. These findings suggest that reduced attention to social information in early childhood may not be specific to ADHD but may instead reflect a broader vulnerability related to neurodevelopmental difficulties. From this perspective, alterations in social gaze during early childhood may represent a developmental characteristic associated with multiple domains of later behavioral and social functioning, including inattention and hyperactivity–impulsivity. Future studies incorporating diagnostic outcomes and measures of functional impairment will be important to clarify whether gaze‐based metrics provide disorder‐specific predictive value or primarily reflect shared developmental pathways across neurodevelopmental conditions.

Regarding sex differences, in the primary complete‐case analyses, significant associations between the small window stimulus and both inattention and hyperactivity–impulsivity symptoms were observed among boys but not among girls. However, sensitivity analyses using IPW showed significant associations for the small window stimulus among girls as well, suggesting that attrition may have contributed to the attenuation of associations among girls in the primary analyses. Although significant associations were also observed among girls after IPW adjustment, the magnitude and consistency of the associations appeared weaker than those observed among boys. Furthermore, in the present cohort, the distribution of percentile scores on the ADHD‐RS was narrower in girls than in boys, which may have reduced the statistical power to detect associations among girls (Supporting Information S3: Table 3). Our previous study using the same cohort (Ref. 38) did not perform sex‐stratified analyses because the focus was on evaluating gaze patterns in a general population context where developmental screening is typically conducted without sex stratification. Therefore, direct comparison of sex‐specific patterns between the two studies is not possible. Nevertheless, the present findings suggest that associations between reduced social gaze and later inattention and hyperactivity–impulsivity may be present in both sexes, while appearing stronger and more consistent among boys.

The present study has some limitations that should be acknowledged. First, information on clinical diagnoses may have been incompletely ascertained because diagnoses were based on caregiver self‐report and some children may never have undergone formal clinical evaluation. Only four children in the analytic sample had a reported physician diagnosis by age 8, a proportion substantially lower than expected based on epidemiologic estimates and lower than the 169 diagnosed children identified in the overall cohort. Therefore, under‐ascertainment of ASD and/or ADHD diagnoses is possible. Furthermore, detailed information on ADHD treatment or intervention status, including medication use, was not available in the current analytic dataset. Second, guardians who suspect that their child has a neurodevelopmental disorder may have more readily chosen answers that tended to reflect this. Therefore, response bias may have existed. Third, the follow‐up rate was low. While 6571 participants completed the detailed survey at age 4, fewer than half (3043 children) completed the ADHD‐RS at age 8. It is possible that a higher proportion of guardians with greater interest in child health and development participated in the follow‐up surveys. Although loss to follow‐up occurred, baseline characteristics were broadly comparable between participants included in the analysis and those excluded because of missing ADHD‐RS data. In addition, sensitivity analyses using IPW yielded results that were generally consistent with those of the primary complete‐case analyses, suggesting that attrition bias is unlikely to fully explain the observed associations. Fourth, rather than being conducted nationwide, the present study was community‐based. However, since ADHD‐RS scores did not substantially differ from nationwide scores, the present findings are likely to have a certain degree of generalizability. Finally, it remains unclear which stimuli assessed with Gazefinder® are related to which characteristics of neurodevelopmental disorders. While the small window stimulus showed the strongest association with inattention traits, other significant associations were also observed (e.g., with silence, still face, talking, and same size stimuli). With Gazefinder®, the selection and utilization of appropriate stimuli for screening remain under discussion.

Despite the limitations above, the present study also has strengths. First, the study was not hospital‐based—it was a large‐scale cohort study that targeted a general population. In addition, the study design was longitudinal and suggested the possibility that the shortness of the duration of eye gaze fixation on social information at age 4, particularly for the small window stimulus, was associated with later inattention and/or hyperactivity–impulsivity symptoms during the early school years. These findings suggest that reduced social gaze in early childhood may represent a developmental characteristic associated with broader neurodevelopmental vulnerability and later behavioral traits, including inattention and hyperactivity–impulsivity symptoms.

## CONCLUSION

The present large‐scale population‐based cohort study is, to our knowledge, the first to demonstrate that shorter eye‐gaze fixation on social information at age 4, as measured using an eye‐tracking system originally developed for ASD detection, is associated with later inattention and hyperactivity–impulsivity symptoms. These findings suggest that early alterations in social gaze may represent a developmental characteristic associated with broader neurodevelopmental vulnerability and later behavioral traits rather than a marker specific to a single disorder.

## AUTHOR CONTRIBUTIONS

Masatsugu Orui conceptualized and designed the study, conducted the initial analyses, drafted the initial manuscript, and revised the manuscript. Shinichi Kuriyama conceptualized, designed, and organized the BirThree Cohort Study and reviewed the manuscript. Masahiro Kikuya, Hirohito Metoki, Taku Obara, Mami Ishikuro, Keiko Murakami, Aoi Noda, and Genki Shinoda managed the implementation of the BirThree Cohort Study and reviewed the manuscript. Kenji J Tsuchiya, Tomoko Nishimura, Yoshihiro Miyake, Keiko Tanaka, Atsushi Hozawa, and Naoki Nakaya reviewed the manuscript critically. All authors approved the final manuscript as submitted and agreed to be accountable for all aspects of the work.

## CONFLICT OF INTEREST STATEMENT

The authors declare no conflicts of interest.

## ETHICAL APPROVAL STATEMENT

This study was approved by the ethics committee of the Tohoku University School of Medicine (approval: 2013‐4‐103; date: May 10, 2013).

## PATIENT CONSENT STATEMENT

N/A.

## CLINICAL TRIAL REGISTRATION

N/A.

## Supporting information

Supporting File 1.

Supporting File 2.

Supporting File 3.

## Data Availability

All data used to support the findings may be released upon application to the Tohoku Medical Megabank Organization.
